# Peptide Sequence Mapping around Bisecting GlcNAc-Bearing *N*-Glycans in Mouse Brain

**DOI:** 10.3390/ijms22168579

**Published:** 2021-08-09

**Authors:** Yuki Ohkawa, Yasuhiko Kizuka, Misaki Takata, Miyako Nakano, Emi Ito, Sushil K. Mishra, Haruna Akatsuka, Yoichiro Harada, Naoyuki Taniguchi

**Affiliations:** 1 Department of Glyco-Oncology and Medical Biochemistry, Osaka International Cancer Institute, 3-1-69 Otemae, Chuo-ku, Osaka 541-8567, Japan; yuki.ohkawa@oici.jp (Y.O.); kizuka@gifu-u.ac.jp (Y.K.); yoharada3@mc.pref.osaka.jp (Y.H.); 2 Center for Highly Advanced Integration of Nano and Life Sciences (G-CHAIN), Gifu University, 1-1 Yanagido, Gifu 501-1193, Japan; lax.runa.0903@gmail.com; 3 Institute for Glyco-Core Research (iGCORE), Gifu University, 1-1 Yanagido, Gifu 501-1193, Japan; 4 Disease Glycomics Team, Global Research Cluster, RIKEN, 2-1 Hisosawa, Wako 351-0198, Japan; emito@ri.ncgm.go.jp; 5 Graduate School of Integrated Sciences for Life, Hiroshima University, 1-3-1 Kagamiyama, Higashihiroshima, Hiroshima 739-8530, Japan; m205138@hiroshima-u.ac.jp (M.T.); minakano@hiroshima-u.ac.jp (M.N.); 6 Glycoscience Group, National University of Ireland Galway, University Road, H91 TK33 Galway, Ireland; skmishra@olemiss.edu

**Keywords:** bisecting GlcNAc, *N*-glycan, mouse brain, glycoproteomics

## Abstract

*N*-glycosylation is essential for many biological processes in mammals. A variety of *N*-glycan structures exist, of which, the formation of bisecting *N*-acetylglucosamine (GlcNAc) is catalyzed by *N*-acetylglucosaminyltransferase-III (GnT-III, encoded by the *Mgat3* gene). We previously identified various bisecting GlcNAc-modified proteins involved in Alzheimer’s disease and cancer. However, the mechanisms by which GnT-III acts on the target proteins are unknown. Here, we performed comparative glycoproteomic analyses using brain membranes of wild type (WT) and *Mgat3*-deficient mice. Target glycoproteins of GnT-III were enriched with E4-phytohemagglutinin (PHA) lectin, which recognizes bisecting GlcNAc, and analyzed by liquid chromatograph-mass spectrometry. We identified 32 *N*-glycosylation sites (Asn-Xaa-Ser/Thr, Xaa ≠ Pro) that were modified with bisecting GlcNAc. Sequence alignment of identified *N*-glycosylation sites that displayed bisecting GlcNAc suggested that GnT-III does not recognize a specific primary amino acid sequence. The molecular modeling of GluA1 as one of the good cell surface substrates for GnT-III in the brain, indicated that GnT-III acts on *N*-glycosylation sites located in a highly flexible and mobile loop of GluA1. These results suggest that the action of GnT-III is partially affected by the tertiary structure of target proteins, which can accommodate bisecting GlcNAc that generates a bulky flipped-back conformation of the modified glycans.

## 1. Introduction

Proteins with *N*- and *O*-glycans are often regulated by post-translational modifications in order to carry out their biological functions in processes such as development, differentiation, metabolism, and cell signaling [1,2]. A number of glycosyltransferases that catalyze the transfer of monosaccharides have been identified in the past few decades, and these enzymes are required for the production of a myriad of glycan chains including *N*- and *O*-glycans [3]. Studies on mice and cell lines with glycosyltransferase mutations have clarified the roles of these glycans and demonstrated that specific glycans modulate protein functions, sometimes leading to disease onset and progression [4,5,6,7].

**Figure 1:**
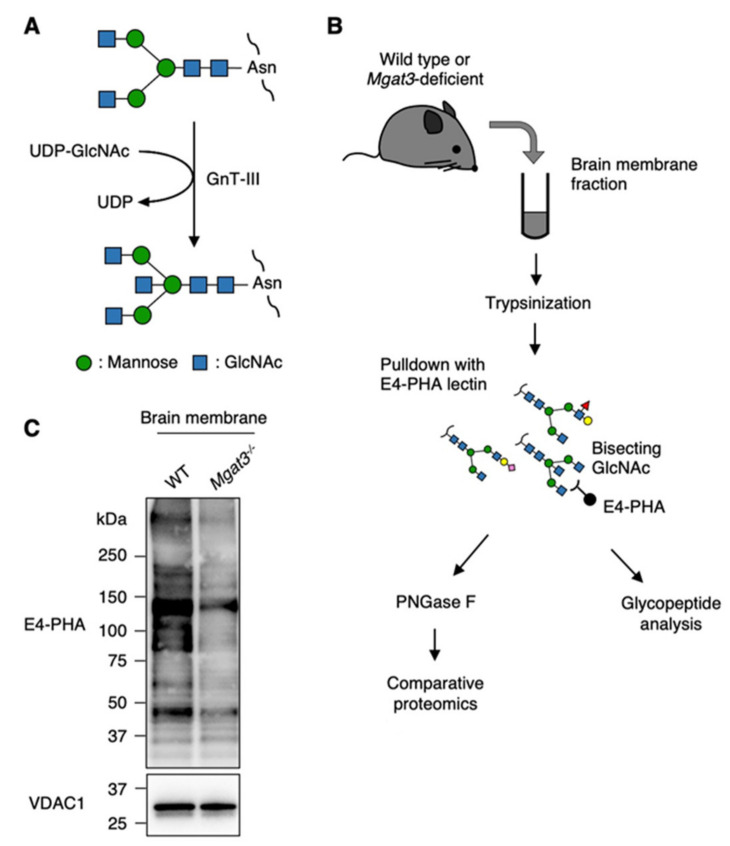
Enrichment of bisecting GlcNAc-bearing proteins with E4-PHA lectin. (**A**) Illustration of the reaction catalyzed by *N*-acetylglucosaminyltransferase-III (GnT-III). (**B**) Experimental design of the present study. Bisecting GlcNAc-bearing glycopeptides from brain membranes of wildtype and *Mgat3*-deficient mice were enriched with E4-phytohemagglutinin (E4-PHA) lectin and analyzed by liquid chromatography-mass spectrometry (LC-MS). (**C**) E4-PHA lectin blot of brain membrane proteins from wildtype and *Mgat3*-decifient (*Mgat3^-/-^*) mice. Western blot with anti-voltage-dependent anion-selective channel 1 (VDAC1) antibody was used as a loading control.

The research undertaken by our group focuses on *N*-acetylglucosaminyltransferase-III (GnT-III, encoded by the *Mgat3* gene), which catalyzes the transfer of *N*-acetylglucosamine (GlcNAc) onto the mannose of an *N*-glycan via a β-1,4-linkage resulting in a bisecting GlcNAc structure (Figure 1A) [8,9,10]. The *Mgat3* gene is highly expressed in the brain and kidney tissues of mammals [11], suggesting that bisecting GlcNAc has physiological and pathological functions in these organs. Based on a previous report which showed *MGAT3* mRNA to be upregulated in brain tissue of patients with Alzheimer’s disease (AD) [12], we investigated the pathological role of bisecting GlcNAc in the brain in the context of AD using *Mgat3*-deficient mice. We found that *Mgat3*-deficient mice exhibit greatly improved AD pathology and improved performance in a memory task. Furthermore, reduced deposition of the disease-causative amyloid-β (Aβ) peptide [13,14] was observed and the β-site amyloid precursor protein cleaving enzyme-1 (BACE1), an Aβ-producing enzyme [15], was found to be heavily modified with bisecting GlcNAc in brain tissues. Moreover, *Mgat3* knockout in the AD model mice caused BACE1 localization to change from the early endosome (the site of Aβ generation) to the lysosome, with consequent suppression of Aβ plaque formation [13]. Importantly, the level of bisecting GlcNAc on BACE1 increases in response to oxidative stress [16], suggesting that Aβ-related oxidative stress in the brain may create a pathological cycle linking bisecting GlcNAc, BACE1, Aβ and oxidative stress. These results strongly suggest that bisecting GlcNAc modification of specific target proteins promotes the onset and progression of AD.

Several research groups reported that GnT-III is involved in tumor malignancy. For example, loss of *Mgat3* in mice caused enhancement of the Ras pathway and promoted tumor progression in the mouse mammary tumor virus (MMTV)-polyoma middle T (PyMT) oncogene-induced mammary gland tumor model [17]. In addition, silencing of *MGAT3* causes cell migration and proliferation, colony formation and epithelial–mesenchymal transition to be enhanced in human breast cancer cell lines [18]. Our research group also reported that mouse melanoma cells that stably express *Mgat3* exhibit weak invasiveness and low lung metastatic ability [19,20,21]. Several glycoproteins with bisecting GlcNAc, including integrins and cadherins, have been reported to mediate these tumor-suppressive phenotypes [20,22,23,24]. In contrast, aberrant upregulation of *MGAT3* and bisecting GlcNAc have also been identified in some cancer cells, promoting tumor malignancy [25,26]. The precise mechanisms underlying these observations have not yet been clarified. Although these findings highlight the importance of GnT-III and its target glycoproteins in cancer biology, the specific bisecting GlcNAc-bearing proteins that are responsible for these tumor-related phenotypes are not yet clear. Recently Taniguchi et al. proposed in epithelial–mesenchymal transition (EMT) programs which play key roles in cancer, significance of GnT-III and other branching enzymes at the intermediate state of EMT [27] and GnT-III acts as EMT as well as its mesenchymal–epithelial transition (MET) as well. This demonstrates the necessity of glycoproteomic analysis to identify target proteins of GnT-III.

Our research group recently reported the presence of bisecting GlcNAc to inhibit various terminal glycan modifications such as sialylation, fucosylation and generation of HNK-1 structure [28]. Several biochemical studies also revealed that other *N*-glycan branching enzymes, such as GnT-IV and GnT-V, do not act on the GnT-III products [29,30,31]. Another research group also reported that the β1,4-galactosyltransferases in the brain did not modify the glycan with bisecting GlcNAc, because bisected *N*-glycans were not used as substrates for β1,4-galactosyltransferases [32]. These studies suggested that altered expression of bisecting GlcNAc led to changes in glycan structure and biological functions of target proteins. Although the importance of the functions of bisecting GlcNAc in AD, cancer and glycan biosynthesis have been reported as described above, the selectivity of GnT-III toward specific target proteins is not fully understood. To understand the mechanisms of GnT-III selectivity, the present study sought to identify proteins which are physiologically modified by GnT-III. To this end, we enriched the bisecting GlcNAc-bearing peptides using a specific plant lectin from mouse brain tissue (where GnT-III is most highly expressed) and performed comparative glycoproteomic analysis of wild type (WT) and *Mgat3*-deficient brains. Moreover, to examine whether bisecting GlcNAc modification requires specific amino acid sequences, we analyzed the peptide sequences in silico around the asparagine residues to which bisecting GlcNAc-bearing *N*-glycans are attached.

## 2. Results

### 2.1. Enrichment of Bisecting GlcNAc-Bearing Proteins with E4-PHA Lectin

Our workflow for the identification of bisecting GlcNAc-bearing proteins from mouse brain tissue is illustrated in Figure 1B. We prepared brain membrane fractions of WT and *Mgat3*-deficient mice. After trypsinization, we enriched the target proteins from brain membrane fractions via pulldown with *Phaseolus vulgaris*-derived E4-phytohemagglutinin (E4-PHA) lectin for proteomic and glycoproteomic analyses (Figure 1B), as this lectin preferentially recognizes bisecting GlcNAc-bearing *N*-glycans [33,34,35] (Supplementary Figure S1). Signals in E4-PHA lectin blots were drastically reduced in brain samples from *Mgat3*-deficient mice (Figure 1C), which confirms the specificity of E4-PHA lectin for bisecting GlcNAc. Notably, the pattern of E4-PHA blots in WT brain indicates that only a limited number of target proteins were modified by GnT-III.

### 2.2. Identification of Bisecting GlcNAc-Bearing Proteins in Brain Tissue by Proteomic Analysis

**Figure 2:**
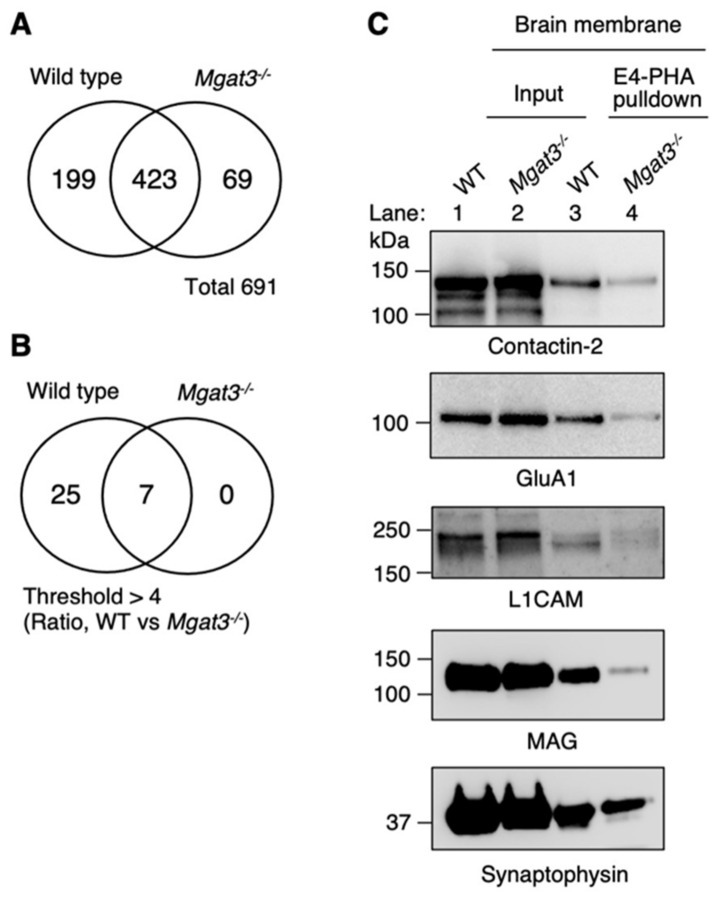
Identification of bisecting GlcNAc-bearing *N*-glycosylation sites in the brain. (**A**) Venn diagram indicating the total number of peptides identified by LC-MS. (**B**) Venn diagram indicating the number of peptides confirmed to be *N*-glycosylated. (**C**) The representative results of Western blot analysis of brain membrane fractions incubated with E4-PHA lectin beads. Membranes were probed with anti-contactin-2, anti-α-amino-3-hydroxy-5-methylisoxazole-4-propionic acid (AMPA)-type glutamate receptor 1 (GluA1), anti-neural cell adhesion molecule L1 (L1CAM), anti-myelin-associated glycoprotein (MAG), and anti-synaptophysin antibodies.

We performed a comparative proteomic analysis of peptides enriched using E4-PHA pulldown from WT and *Mgat3*-deficient mouse brain samples. Prior to proteomic analysis, *N*-glycans were removed from the glycopeptides using peptide *N*-glycosidase F (PNGase F) [36], resulting in the conversion of Asn (N) residues to Asp (D), which is the trace of *N*-glycosylation from the original peptides. We identified 691 peptides in total from WT and *Mgat3*-deficient brain samples (Figure 2A and Supplementary Table S1). Among these, we identified 32 peptides that were *N*-glycosylated, as indicated by N to D conversion (Figure 2B). Out of the 32 peptides, 21 were detected in WT samples only, and the rest of the peptides were more abundant in WT than *Mgat3*-deficient samples (Table 1 and Figure 2B) (4 peptides, WT vs. KO > 4; 7 peptides, 4 > WT vs. KO > 1). This strongly suggests that 32 sites were indeed modified by GnT-III to express bisecting GlcNAc. Among the identified 32 peptides, we analyzed the expression of bisecting GlcNAc on contactin-2, α-amino-3-hydroxy-5-methylisoxazole-4-propionic acid (AMPA)-type glutamate receptor 1 (GluA1), neural cell adhesion molecule L1 (L1CAM), myelin-associated glycoprotein (MAG), and synaptophysin by biochemical analysis using lectin pulldown and Western blots. All these proteins were pulled down with E4-PHA from membrane extracts of WT brain samples (Figure 2C, third lane), while the signals from *Mgat3*-deficient brain samples were reduced (Figure 2C, forth lane), confirming that these proteins are modified with bisecting GlcNAc in brain tissues.

### 2.3. LC-MS Analysis of a Bisecting GlcNAc-Bearing Glycopeptide in the Brain

**Figure 3:**
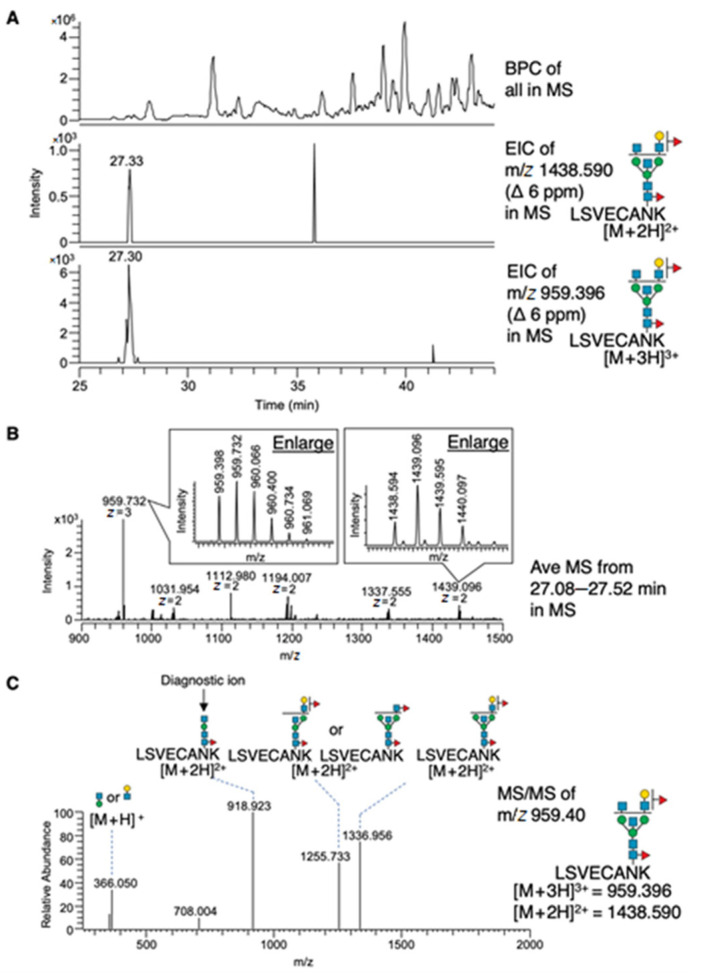
LC-MS and MS/MS analysis of bisecting GlcNAc-bearing glycopeptides in the brain. (**A**) MS chromatograms of glycopeptides enriched by E4-PHA lectin from wildtype brain membranes. Base peak chromatogram (upper panel). Extracted ion chromatogram (EIC) for ions with masses calculated as [M + 2H]^2+^ and [M + 3H]^3+^ of a bisecting GlcNAc-bearing glycopeptide derived from synaptophysin is selectively shown (middle and lower panels), which was confirmed by tandem mass spectrometry to be modified with bisecting GlcNAc. (**B**) Average MS spectrum around the peak (27 min) detected in middle and lower panels of (A). Enlargements show that the monoisotopic ions were the corresponding glycopeptides on accurate mass and charge state. (**C**) MS/MS spectrum of the [M + 3H]^3+^ parent ion is shown in the lower chromatogram of (**A**). The N-glycan structure of each peak was deduced as indicated.

To further confirm that bisecting GlcNAc-bearing N-glycans are attached onto the identified brain proteins, we analyzed glycopeptides by LC-MS. We prepared trypsin-digested glycopeptides from WT brain membrane fractions and enriched the target glycopeptides with E4-PHA lectin similarly to the above proteomic analysis. The enriched glycopeptides were then analyzed by LC-MS/MS without PNGase treatment (Figure 3A, upper panel). Among the identified peptides in Table 1, we focused on the peptide derived from synaptophysin (LSVECANK). We manually searched for the peptide that is modified with one of the most abundant bisected N-glycans in mouse brain [28], and its extracted ion chromatogram (EIC) is shown (Figure 3A, middle and lower panels). The average MS spectrum at a retention time between 27.08 and 27.52 min confirmed the presence of the [M + 2H]^2+^ and [M + 3H]^3+^ ions of glycopeptide (Figure 3B). MS/MS analysis of the glycopeptide detected a reliable diagnostic ion for bisecting GlcNAc (m/z 918.923) (Figure 3C), confirming that bisecting GlcNAc is indeed present on the identified peptide of synaptophysin.

### 2.4. Peptide Sequence Map around Bisecting GlcNAc-Bearing N-Glycans in the Brain

**Figure 4:**
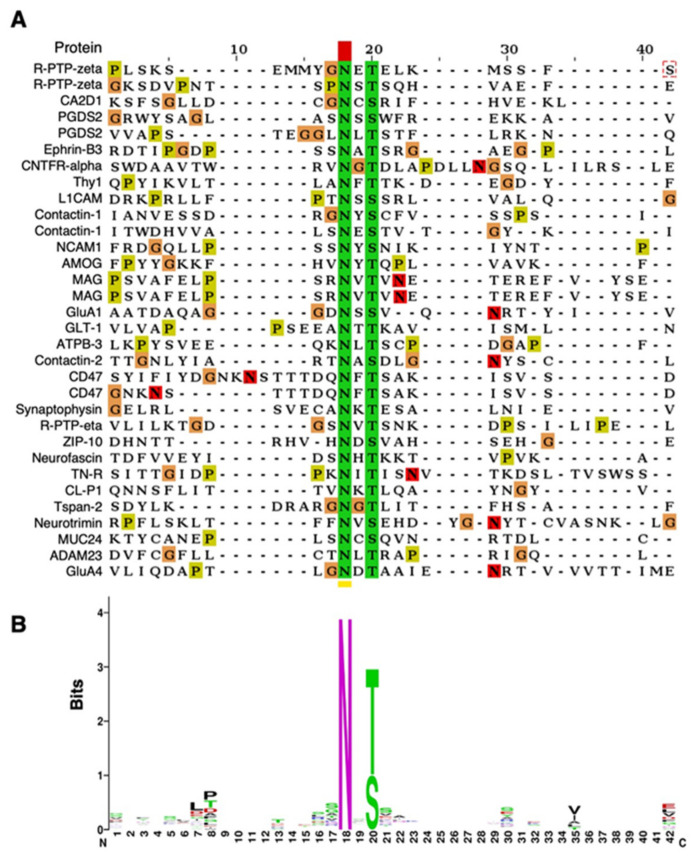
Peptide sequence map around bisecting GlcNAc-bearing *N-*glycans in mouse brains. (**A**) Multiple sequence analysis of glycopeptide sequences and their *N-*glycosylation sites of the identified peptides. (**B**) The consensus sequence of the aligned glycopeptides is shown as WebLogo.

Sequence alignment of the identified 32 peptides (Figure 4A) showed asparagine residues (indicated in green) that were identified to be highly potential *N*-glycosylation sites with bisecting GlcNAc. As Figure 4B illustrates, no unique amino acids or motifs were identified around the modified sites. This result suggests that GnT-III does not recognize a specific primary amino acid sequence to transfer bisecting GlcNAc, raising the possibility that the action of GnT-III may be regulated by the tertiary structure of target proteins.

### 2.5. Structural Model of GluA1 Docked with N-Glycans

**Figure 5:**
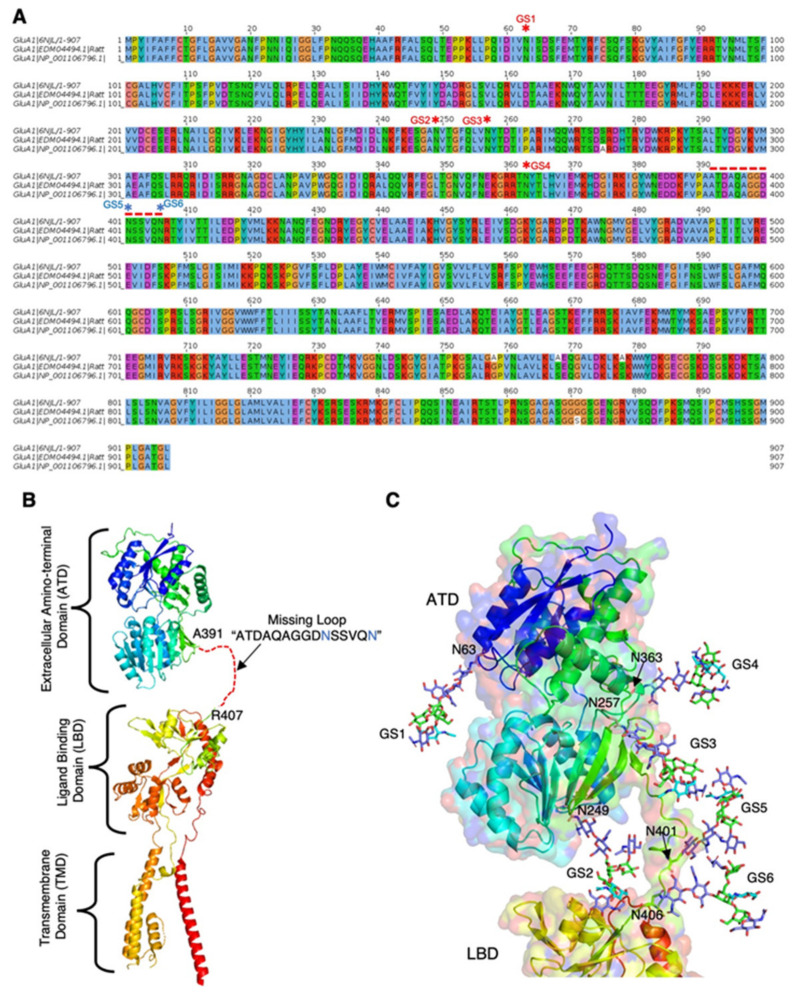
Sequence and structural model of GluA1 receptor. (**A**) Comparison of GluA1 receptor amino-acid sequence among the crystal structure (PDB ID: 6NJL; organism: *Rattus norvegicus*), Genbank sequence from *Rattus norvegicus* (GenBank ID: EDM04494.1) and from *Mus musculus* (NP_001106796.1). Asterisks represent glycosylated Asn. (**B**) Full-length structure of GluA1 receptor (PDB ID: 6NJL). A missing loop region (A392-TDAQAGGD-N401-SSVQN406) is shown with dotted red lines. (**C**) Six glycosylation sites (Asn63, Asn249, And257, Asn363, Asn401, and Asn406) are labeled as GS1 to GS6. The GlcNAc (blue), bisecting GlcNAc (cyan), and Man (green) are shown as sticks.

To further investigate how GnT-III selectively acts on target proteins, we finally generate a structural model of one of the typical identified target proteins like GluA1. Since there are not sufficient crystal structures having bisecting *N*-glycans, it is impossible to look for geometrically critical residues for bisecting GlcNAc modification. We modeled *N*-glycans in GluA1 receptor to see if the identified *N*-glycosylation site can be accessed by the GnT-III for bisecting GlcNAc transfer (Figure 5). 3D structure of GluA1 was extracted from the complex structure of rat AMPA receptor recently solved by cryo-EM [37]. Sequence comparison of GluA1 among the solved structure, rat GluA1 and mouse GluA1 in GenBank database confirmed that all of 6 *N*-glycosylation sites were conserved between rat and mouse GluA1 (PDB: 6NJL) (Figure 5A). The overall structure of GluA1 comprises amino-terminal domain (ATD), ligand binding domain (LBD), and transmembrane domain, and there is a loop between ATD and LBD which is missing in the structure (Figure 5B). GluA1 has six *N*-glycosylation sites (Figure 5A,C, GS1 to GS6), and the glycopeptide containing the 5th (GS5, Asn401) and 6th (GS6, Asn406) sites were identified in our proteomic analysis, indicating that either GS5 or GS6 or both sites can be modified by GnT-III. As the bisected *N*-glycans occupy a larger space than biantennary glycans [28,35], it was expected that GnT-III acts on *N*-glycans located in the relatively unconstrained regions. In a modeled structure of GluA1, bisected *N*-glycans were successfully fitted to all 6 *N*-glycosylation sites of the protein without an apparent steric clash (Figure 5C). Furthermore, a hydroxyl group at the C3 position of the acceptor Man residue is exposed nicely in five out of six glycosylation sites, allowing GnT-III to add a GlcNAc residue. In particular, *N*-glycans at GS5 and GS6 are located in the mobile loop and highly flexible, which is likely one of the preferred sites for modification by GnT-III. In contrast, the glycosylation site at Asn249 (GS2) is pointing towards the ligand binding domain and might not be accessible to GnT-III. Together, these results suggest that the tertiary structure of acceptor proteins is a possible mechanism that determines the acceptor specificity of GnT-III.

## 3. Discussion

The present study utilized comparative glycoproteomic analyses to identify bisecting GlcNAc-bearing proteins in mouse brain samples. Non-specific binding of lectin is a common problem in glycoproteomic analyses, and the analysis of control samples from glycosyltransferase-deficient mice is often used to overcome this issue [38]. Therefore, we used *Mgat3*-deficient brain tissue as a negative control to enable reliable identification of the protein targets of GnT-III.

We identified in total 32 *N*-glycosylation sites as in vivo targets of GnT-III. Almost all the identified sites (31 out of 32) were also included in a previous *N*-glycoproteomic study of mouse brain reported by Zielinska et al. [39]. However, sequence alignment did not identify any diagnostic amino acid or motif around these sites (Figure 4A,B). This suggests that the specificity of bisecting GlcNAc modification is not dictated by the primary sequence, but by other factors such as tertiary motifs of acceptor proteins or the precise sub-Golgi localization of GnT-III. The mechanism by which glycosyltransferase acts on target proteins is poorly understood. Structural analyses of target glycoproteins or detailed studies of the sub-Golgi localization of GnT-III could provide insight into the differential modification of glycoproteins with various glycan structures. Our structural models of GluA1 show that the identified sites for bisecting GlcNAc modification are located in a highly mobile and flexible region, highlighting the role of tertiary structure of acceptor proteins in the substrate specificity of GnT-III. Docking models of acceptor proteins with various glycosyltransferases including GnT-III will provide deeper insights into important aspects of substrate specificity of glycosyltransferases.

Notably, once GnT-III modifies an *N*-glycan, many glycosyltransferases including GnT-IV, GnT-V, sialyltransferases and fucosyltransferases show greatly reduced activity toward the glycan [29,40,41,42]. In particular, GnT-V, which transfers GlcNAc to the OH-6 position of α-linked mannose and is highly related to tumor metastasis [43,44,45,46], cannot transfer GlcNAc to bisected glycans. Our recent structural study showed that the catalytic pocket of GnT-V would cause a steric clash with a bisecting GlcNAc on acceptor glycans [47]. Considering that GnT-V-producing glycans are involved in tumor malignancy, the regulation of bisecting GlcNAc biosynthesis could influence other steps of *N*-glycan biosynthesis and, therefore, glycan-related phenotypes. Furthermore, the donor sugar nucleotide, uridine diphosphate (UDP)-GlcNAc, is a substrate of all GlcNAc transferases, including GnT-III. Therefore, the up- or down-regulation of one GlcNAc transferase could indirectly influence the activity of other GnTs by altering the available UDP-GlcNAc.

Several reports demonstrated the physiological or pathological roles of *N*-glycans on the proteins identified in the present study. The AMPA-type glutamate receptors are crucial for excitatory postsynaptic transmission and synaptic plasticity of neurons [48]. Four subunits, GluA1, GluA2, GluA3 and GluA4, form a homo- or hetero-complex, and complex formation and localization at the postsynaptic density are critically important for their functions in synaptic transmission. Notably, several research groups reported that deficiency of *N*-glycan at specific glycosylation sites of GluA1 and GluA2 impair cell-surface expression and complex formation of the subunits [49,50], suggesting that proper *N*-glycosylation of these subunits is required for synaptic activity. The importance of *N*-glycans on contactin-1 and -2 were also reported. The *N*-glycans on contatin-1 have been described to be involved in chronic inflammatory demyelinating polyradiculoneuropathy (CIDP) and Guillain-Barré syndrome [51]. Site-specific *N*-glycosylation of contactin-1 is involved in autoimmune targeting, and the recognition of contactin-1 with oligomannose-type *N*-glycans by autoantibodies from patients with CIDP leads to an enhanced disease state. Contactin-2 (also designated as TAG-1/Axonin-1) is known to play significant roles in cell migration, axonal polarization, and myelination through interactions with various molecules, including cell adhesion molecules, neuropilin-1, and Caspr2 [52]. Moreover, lectin activity of contactin-2 was also reported, and its specific binding to a brain-type glycan structure containing Lewis X epitope regulates neurite outgrowth [53]. However, the functions of bisecting GlcNAc on these molecules have not yet been demonstrated since bisecting GlcNAc was found to regulate the subcellular localization and activity of various carrier proteins [13,23], and *Mgat3*-deficient mice showed the neurological phenotypes with behavioral abnormality [54]. The future functional analyses of the identified molecules in the brain of *Mgat3*-deficient organisms could lead to the elucidation of novel functions of bisecting GlcNAc in the brain.

## 4. Materials and Methods

### 4.1. Mice

*Mgat3*-knockout mice were generated and generously provided by Dr. Jamey D. Marth (University of California, Santa Barbara) [55]. We used WT and *Mgat3*-deficient mice (C57BL/6 genetic background). All animal experiments were approved by the Animal Experiment Committee of RIKEN and Gifu University.

### 4.2. Preparation of Brain Membrane Fractions

Mouse brains were homogenized in seven volumes of Tris-buffered saline (TBS) containing a protease inhibitor cocktail (Roche; Basel, Switzerland) using a Potter-type tissue grinder. After removal of the nuclear fraction and unbroken cells by low-speed centrifugation (500× *g* for 10 min at 4 °C), the supernatant was ultracentrifuged at 105,000× *g* for 30 min at 4 °C. The resulting pellet was used as the total membrane fraction for lectin pulldown, and Western and lectin blotting.

### 4.3. Pull down with E4-Phytohemagglutinin Lectin

The total membrane fraction derived from 250 µL of brain homogenate supernatant was solubilized in 1 mL of TBS containing 1% (*v/v*) Triton X-100 and protease inhibitor cocktail. After centrifugation at 20,000× *g* for 15 min at 4 °C, the supernatant was incubated with E4-PHA-agarose (EY Laboratories, San Mateo, CA, USA) overnight at 4 °C with gentle shaking. After washing the beads twice with TBS containing 0.1% (*v/v*) Triton X-100, bound proteins were eluted by boiling with Laemmli sample buffer.

### 4.4. Western and Lectin Blots

Proteins were separated by 5–20% (*w/v*) SDS-PAGE, then blotted onto a nitrocellulose membrane. For Western blot, membranes were blocked with 5% (*w/v*) skimmed milk in TBS containing 0.1% (*v/v*) Tween 20 (TBS-T), followed by incubation with primary and horseradish-peroxidase (HRP)-conjugated secondary antibodies. The primary antibodies used for this study were: anti-voltage-dependent anion-selective channel 1 (anti-VDAC1; ab14734, Abcam, Cambridge, UK), anti-Contactin-2 (AF4439, R&D, Minneapolis, MN, USA), anti-GluA1 (ab109450, Abcam) and anti-L1CAM (ab24345, Abcam). For lectin blotting, membranes were blocked with TBS-T, then incubated with HRP-conjugated E4-PHA, biotinylated AAL (J201R, J-chemical, Tokyo, Japan) or ConA (J203, J-chemical, Tokyo, Japan). Biotinylated lectins were detected with VECTASTAIN ABC kit (Vector Laboratories, Burlingame, CA, USA). Conjugation of E4-PHA (J111, J-chemical, Tokyo, Japan) with HRP was performed using a peroxidase labeling kit NH2 (DOJINDO, Kumamoto, Japan) according to the manufacturer’s protocol. Briefly, 200 µg of E4-PHA (2 mg/mL in phosphate-buffered saline) was diluted with 100 µL of wash buffer (supplied in the kit), then the solution was applied to a filtration tube supplied in the kit and centrifuged at 8000× *g* for 10 min. After this, 10 µL of reactive HRP solution (supplied in the kit) was added to the column, followed by incubation for 2 h at 37 °C. Wash buffer (100 µL) was added to the column, and the tube was centrifuged at 8000× *g* for 10 min. Storage buffer (200 µL, supplied in the kit) was applied to the column, and labeled E4-PHA was eluted by pipetting 10 times. Two or three independent experiments were performed to confirm the reproducibility, and the representative images were shown in the figures.

### 4.5. Liquid Chromatography-Mass Spectrometry for Glycan Structural Analysis

Three brains from adult WT mice were homogenized in 10 mM HEPES pH 7.5 with protease inhibitor cocktail (Roche), followed by incubation for 1 h on ice. After removal of nuclei and cell debris by centrifugation (500× *g*, 4 °C, 10 min), the supernatant was ultracentrifuged at 105,000× *g* at 4 °C for 1 h to pellet the membranes. The membrane fraction containing 3 mg proteins was suspended in 500 µL of a reducing solution containing 250 mM Tris-HCl (pH 8.5), 6 M guanidine hydrochloride, 2 mM EDTA and 11 mg of dithiothreitol (DTT), and then was incubated at 50 °C for 1 h. The reduced proteins were alkylated with 40 mg of iodoacetamide (IAA) by incubating at room temperature for 1 h in the dark. The reaction mixture was pass through a PD-10 column (GE Healthcare, Chicago, IL, USA) equilibrated with 10 mM NH_4_HCO_3_ to remove salts from the reducing solution and excess IAA. The desalted solution containing S-carbamidomethylated proteins was evaporated to dryness. The dried residue was dissolved with 500 µL of 50 mM NH_4_HCO_3_ containing 20 µg trypsin (Thermo Fisher Scientific, Boston, MA, USA), and then was incubated at 37 °C for 16 h. After boiling to inactivate trypsin, 10 µg of biotinylated E4-PHA dissolved in 100 µL of a solution containing 500 mM Tris-HCl (pH 7.4) and 1 M NaCl was added to the digestive solution, and the sample was gently agitated overnight at 4 °C. One mg of Dynabeads M-280 Streptavidin (Thermo Fisher Scientific, Boston, MA, USA) suspended in 50 µL of a solution containing 42 mM NH_4_HCO_3_, 83 mM Tris-HCl (pH 7.4) and 167 mM NaCl was added to the solution, and the sample was gently agitated for 2 h at 4 °C. After discarding the solution that was not attracted by magnet, an elution buffer containing 40 mM NH_4_HCO_3_, 4 M urea and 4 mM DTT was added to the residue attracted by magnet, and the bound proteins were eluted by incubation at 50 °C for 1 h. Glycopeptides were desalted using NuTip Carbon (Glygen, Columbia, MD, USA).

Glycopeptides were dissolved in 20 μL of 0.08% (*v/v*) formic acid. Glycopeptides were separated using an ODS column (Develosil 300ODS-HG-5; 150 × 1.0 mm ID; Nomura Chemicals, Japan) under specific gradient conditions. The mobile phases were solvent A (0.08% (*v/v*) formic acid) and solvent B (0.15% (*v/v*) formic acid in 80% (*v/v*) acetonitrile). The column was eluted with solvent A for 5 min, at which point the concentration of solvent B was increased to 40% (*v/v*) over 55 min at a flow rate of 50 μL/min using an Accela HPLC system (Thermo Fisher Scientific, Boston, MA, USA). The eluate was introduced continuously into an ESI source, and the glycopeptides were analyzed by LTQ Orbitrap XL (hybrid linear ion trap-orbitrap mass spectrometer; Thermo Fisher Scientific). In the MS setting, the voltage of the capillary source was set at 4.5 kV, and the temperature of the transfer capillary maintained at 300 °C. The capillary voltage and tube lens voltage were set at 15 and 50 V, respectively. MS data were obtained in positive ion mode over the mass range *m/z* 300 to *m/z* 3000 (resolution: 60000, mass accuracy: 6 ppm). MS/MS data were obtained by ion trap (top three in mass list of parent ions, CID).

### 4.6. Proteomic Analysis

Total membrane proteins from WT and *Mgat3^-/-^* mouse brains were prepared (4 mice for each genotype were pooled for preparation fo the membrane fractions). After the addition of 600 µL of 6 M guanidine HCl/100 mM NH_4_HCO_3_ to 3 mg of the total membrane proteins (1.4 mg/mL), DTT was added (final 5 mM), followed by incubation for 1 h at 37 °C. IAA was added (final 20 mM), followed by incubation for 1 h at room temperature. After desalting using a PD10 column, protein concentrations were measured using BCA assay. The proteins (80 µg/200 µL × 10 tubes) were dried and dissolved in 200 µL (per tube) of 25 mM NH_4_HCO_3_ pH 8.0 containing 4 mg (per tube) of Trypsin Gold (Promega), followed by overnight incubation at 37 °C. Five volumes of ice-cold acetone were added, and the samples were incubated for 20 h at −20 °C to precipitate peptides. After centrifugation at 12,000× *g* for 10 min, peptides were dissolved in 1 mL of 10 mM NH_4_HCO_3_, 150 mM NaCl, 5 mM CaCl_2_ and 5 mM MgCl_2_. Biotinylated E4-PHA (60 µg) was added to the sample, and the mixture incubated overnight at 4 °C. Dynabeads M-280 Streptavidin (1 mg) was added, followed by further incubation for 2 h at 4 °C with gentle shaking. The beads were washed three times with a buffer containing 10 mM NH_4_HCO_3_, 150 mM NaCl, 5 mM CaCl_2_ and 5 mM MgCl_2_, and the bound peptides eluted by incubation for 1 h at 50 °C in a buffer containing 40 mM NH_4_HCO_3_, 4 M urea and 4 mM DTT. Glycopeptides were desalted using NuTip Carbon (Glygen, Columbia, MD). NuTip was pretreated sequentially with solution A (90% (*v/v*) acetonitrile and 0.5% (*v/v*) formic acid) and with solution B (0.5% (*v/v*) formic acid). The peptides bound to NuTip were washed with solution B and then eluted with solution A, followed by treatment with 2 U of PNGaseF (Roche) for 37 °C overnight. The peptides treated with PNGaseF were purified using NuTip Carbon, desalted with ZipTip (Millipore, Burlington, MA, USA), and subjected to proteomic analysis.

The tryptic peptides were trapped using a short ODS column (PepMap 100; 5 μm C18, 20 mm × 100 μm ID; Thermo Fisher Scientific) and then separated with another ODS column (Nano HPLC Capillary Column; 3 μm C18, 150 mm × 75 μm ID; Nikkyo Technos, Tokyo, Japan) using nano-liquid chromatography (EASY-nLC 1000; Thermo Fisher Scientific). The mobile phases for separation were solvent A (0.1% (*v/v*) formic acid in water) and solvent B (0.1% (*v/v*) formic acid in acetonitrile). After loading tryptic peptides onto the trap column with 0.1% (*v/v*) trifluoroacetic acid in 2% (*v/v*) acetonitrile, the concentrated tryptic peptides were eluted from the trap column and separated on the separation column using two-step linear gradients elution: 0–100 min, 0–30% (*v/v*) solvent B; 100–120 min, 30–65% (*v/v*) solvent B at a flow rate of 300 nl/min. The eluate from the separation column was continuously introduced into a nanoESI source and analyzed by mass spectrometry (MS) and MS/MS (Q Exactive; Hybrid Quadrupole-Orbitrap, Thermo Fisher Scientific, Boston, MA, USA).

The MS and MS/MS spectra were generated in the positive ion mode (mass range: m/z 350 to 1800, data-dependent MS2 scan of the top 10 peaks using HCD). The voltage of the capillary source was set at 2.3 kV, and the temperature of the transfer capillary was maintained at 275 °C. The S-lens RF level was set at 55. All MS/MS data were searched using software Proteome Discoverer (version 1.4, Thermo Fisher Scientific) with the MASCOT search engine (ver. 2.5.1, Matrix Sciences, Boston, MA, USA). Doubly, triply, quadruply, and quintuply charged peptide ions were subjected to the database search with a parent and fragment ion mass tolerance of ±6 ppm and ±20 mmu, respectively. Possible static and chemical modifications included were cysteine carbamidomethylation, methionine oxidation and deamidation of asparagine and glutamine. Taxonomy was set to Mus musculus (house mouse) in Swiss Prot 2016_02. Peptide FDR was set at <5% and one missed trypsin cleavage was allowed.

The identified peptides are listed in Supplementary Table S1 (yellow, peptides only detected from WT sample; blue, peptides with signal intensity ratio (WT to KO) >2; light blue, peptides with signal intensity ratio (WT to KO) <2; orange, peptides only detected from KO sample).

### 4.7. Search for Common Sequence around Bisecting N-Acetylglucosamine-Containing Sites

Glycopeptide sequences were aligned by the multiple sequence alignment program T-coffee available on the EMBL-EBI webpage [56]. All sequence alignment parameters were set to default values. The consensus sequence was obtained by calculating the frequency of each amino acid in each position of the sequence of the aligned sequences using the Weblogo tool [57].

### 4.8. Structural Model

A bisected biantennary *N*-glycan was modeled on six different glycosylation sites of GluA1 receptor. 3D structure of GluA1 receptor was extracted from the native AMPA-receptor complexes [37] from Protein Data Bank (PDB ID: 6NJL). The cryoEM structure of GluA1 carries only chitobiose core of the *N*-glycans at four different glycosylation sites (Asn52, Asn242, And250 and Asn356), but two glycosylation sites in the loop region between ATD and LBD are missing. The missing loop “ATDAQAGGDNSSVQN” was modeled using program SuperLooper2 [58]. Conformation of the loop region was adopted from the structure of glycogen phosphorylase (PDB ID: 3DDS). Structure and conformation of *N*-glycan were taken from the biantennary *N*-glycan reported in the structure of catrocollastatin/VAP2B (PDB ID: 2DW2) [59]. Bisected biantennary *N*-glycans were modeled by fitting GlcNAc-1 ring atoms of the model to the one reported in the structure using PyMol (https://pymol.org/2/). Bisected biantennary *N*-glycans in Asn401 and Asn406 were modeled manually on predicted orientation of Asn residues. Protein sequences of GluA1 from the cryoEM structure, rat (GenBank: EDM04494.1) and mouse (NCBI ref. seq: NM_001113325.2) were aligned using EMBL-EBI tools.

## 5. Conclusions

In the post-genomic era, studies of post-translational modifications have become increasingly important. The technology available for the analysis of protein glycosylation is growing rapidly [60]. Physiological glycosylation of proteins is critical for many cell functions; hence, alteration of glycan structure is associated with disease onset and progression. Thus, clarification of the molecular mechanisms underlying the disease-associated alteration in glycosylation will open an avenue for drug discovery and design for diseases that are currently difficult to treat.

In the present study, we identified 32 bisecting GlcNAc-bearing peptides in the mouse brain, including GluA1, contactin-2, L1CAM, MAG, and synaptophysin. Sequence alignment of the identified proteins was unable to identify any diagnostic primary amino acid sequences around the sequon, raising a possibility that GnT-III recognizes the tertiary structure, but not a primary amino acid motif, of the acceptor proteins to add bisecting GlcNAc. Our data on the modeling of GluA1 as a typical substrate for GnT-III suggests that GnT-III acts on *N*-glycosylation site within a highly flexible and mobile loop in GluA1, indicating that GnT-III may prefer *N*-glycosylation sites located in a relatively unconstrained region of the acceptor proteins.

## Figures and Tables

**Table 1:** List of identified bisecting GlcNAc-bearing proteins in mouse brains.

No.	Peptide Sequence Identified	Protein Name	Short Name or Alternative Name	Ratio of Signal Intensity (WT/KO)
1	nITISnVTK	Tenascin-R	TN-R	4.14
2	nVTVnETER	Myelin-associated glycoprotein	MAG	2.44
3	VLTLAnFTTK	Thy-1 membrane glycoprotein	Thy1	3.63
4	LLFPTnSSSR	Neural cell adhesion molecule L1	L1CAM	WT only
5	TGDGSnVTSnFTK	Receptor-type tyrosine-protein phosphatase eta	R-PTP-eta	WT only
6	nSTTTDQnFTSAK	Leukocyte surface antigen CD47	CD47	12.68
7	DTIPGDPSSnATSR	Ephrin-B3	-	WT only
8	SEMMYGnETELK	Receptor-type tyrosine-protein phosphatase zeta	R-PTP-zeta	WT only
9	VLVAPPSEEAnTTK	Excitatory amino acid transporter 2	GLT-1	4.59
10	SFSGLLDcGncSR	Voltage-dependent calcium channel subunit alpha-2/delta-1	CA2D1	WT only
11	FHVnYTQPLVAVK	Sodium/potassium-transporting ATPase subunit beta-2	AMOG	WT only
12	DGQLLPSSnYSNIK	Neural cell adhesion molecule 1	NCAM1	1.3
13	GnYScFVSSPSITK	Contactin-1	-	2.66
14	WYSAGLASnSSWFR	Prostaglandin-H2 D-isomerase	PGDS2	WT only
15	ETLqNNSFLITTVnK	Collectin-12	CL-P1	WT only
16	LSVEcAnK	Synaptophysin	-	WT only
17	HDVFcGFLLcTnLTR	Disintegrin and metalloproteinase domain-containing protein 23	ADAM23	WT only
18	TYcANEPLSncSqVNR	Sialomucin core protein 24	MUC24	WT only
19	TVVAPSTEGGLnLTSTFLR	Prostaglandin-H2 D-isomerase	PGDS2	WT only
20	FVPAATDAQAGGDnSSVQnR	Glutamate receptor 1	GluA1	WT only
21	VnGTDLAPDLLnGSQLILR	Ciliary neurotrophic factor receptor subunit alpha	CNTFR-alpha	WT only
22	ARGnGTLITFHSAFQccGK	Tetraspanin-2	Tspan-2	WT only
23	LVLIQDAPTLGnDTAAIEnR	Glutamate receptor 4	GluA4	WT only
24	nLTScPDGAPFIQHGPDYR	Sodium/potassium-transporting ATPase subunit beta-3	ATPB-3	WT only
25	HNFRPGTDFVVEYIDSnHTK	Neurofascin	-	WT only
26	YIITWDHVVALSnESTVTGYK	Contactin-1	-	2.66
27	TnASDLGnYScLATSHLDFSTK	Contactin-2	-	WT only
28	SDVPNTSPnSTSQHVAEFETER	Receptor-type tyrosine-protein phosphatase zeta	R-PTP-zeta	WT only
29	LTFFnVSEHDYGnYTcVASNK	Neurotrimin	-	3.08
30	SNPEPSVAFELPSRnVTVnETER	Myelin-associated glycoprotein	MAG	2.44
31	SYIFIYDGnKNSTTTDQnFTSAK	Leukocyte surface antigen CD47	CD47	12.68
32	HVHnDSVAHSEHGEPGHSPSPETnK	Zinc transporter ZIP10	ZIP-10	WT only

## Data Availability

The data presented in this study is available on request from the corresponding author.

## Supplementary Materials

The following are available online at https://www.mdpi.com/article/10.3390/ijms22168579/s1, Figure S1: Specificity of E4-PHA pulldown, Table S1: List of all identified peptides in comparative proteomics.
