# Supplementary material for: Peptide Sequence Mapping around Bisecting GlcNAc-Bearing N-Glycans in Mouse Brain
Source: Int J Mol Sci. 2021 Aug 9;22(16):8579. doi: 10.3390/ijms22168579 (PMC8395275; doi:10.3390/ijms22168579)
Supplement: Supplementary file 1 [file ijms-22-08579-s001.zip › Submission/Supplementary Figures_.pptx]

## Slide 1
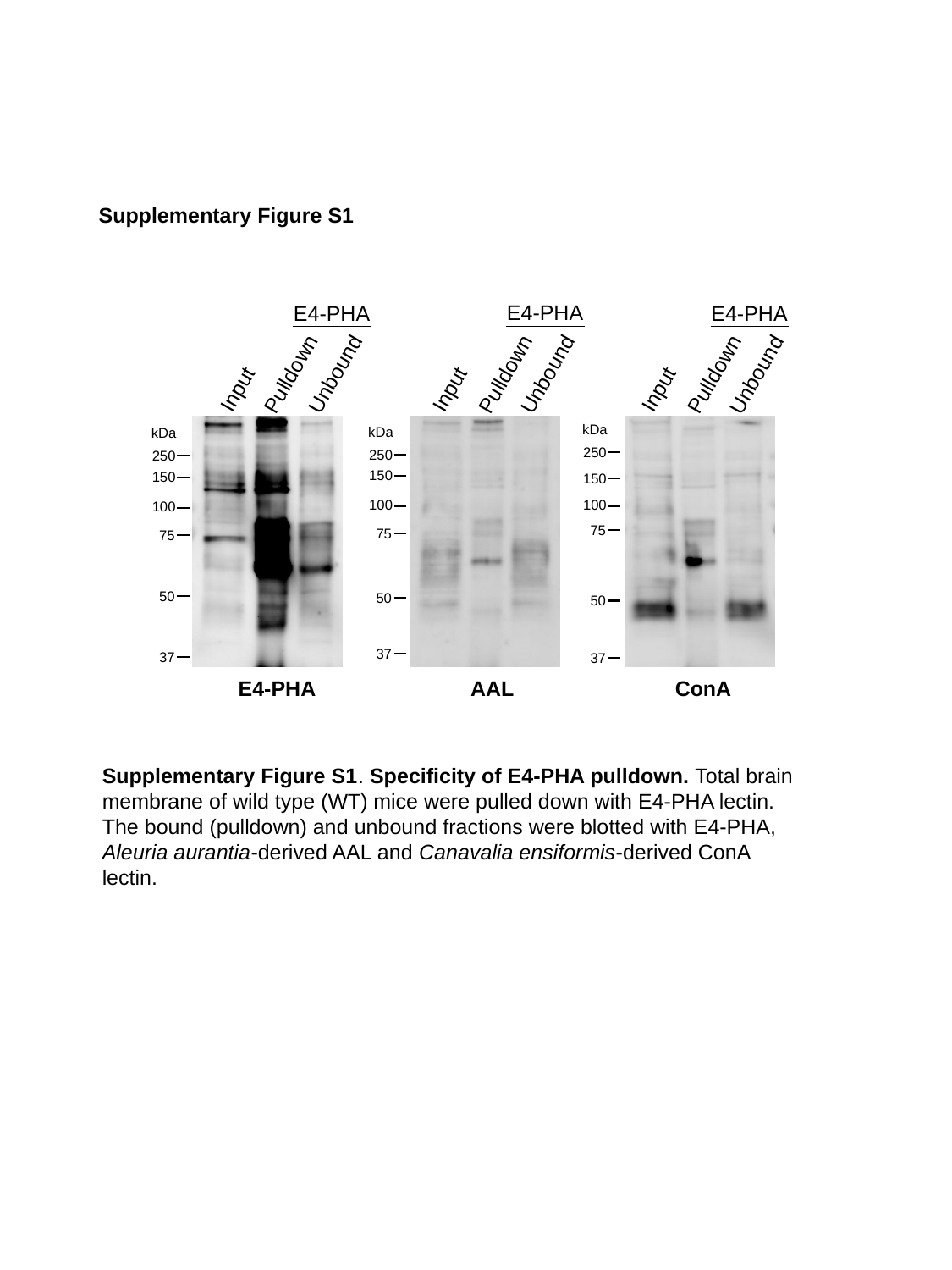

Supplementary Figure S1
E4-PHA
E4-PHA
E4-PHA
Pulldown
Unbound
Pulldown
Unbound
Pulldown
Unbound
Input
Input
Input
kDa
kDa
kDa
250
250
250
150
150
150
100
100
100
75
75
75
50
50
50
37
37
37
E4-PHA
AAL
ConA
Supplementary Figure S1. Specificity of E4-PHA pulldown. Total brain membrane of wild type (WT) mice were pulled down with E4-PHA lectin. The bound (pulldown) and unbound fractions were blotted with E4-PHA, Aleuria aurantia-derived AAL and Canavalia ensiformis-derived ConA lectin.

## Slide 2
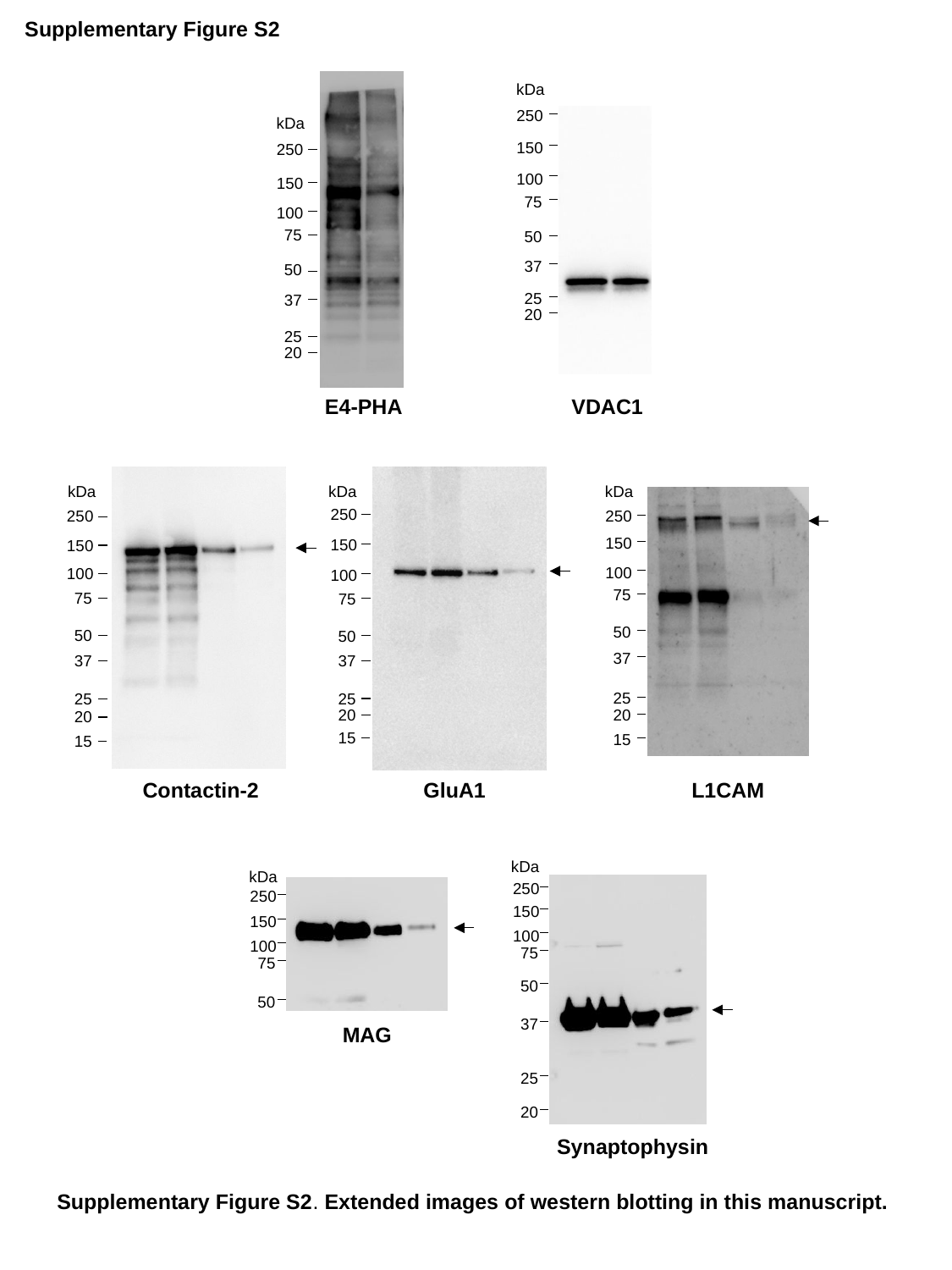

Supplementary Figure S2
kDa
250
kDa
150
250
100
150
75
100
75
50
37
50
25
37
20
25
20
E4-PHA
VDAC1
kDa
kDa
kDa
250
250
250
150
150
150
100
100
100
75
75
75
50
50
50
37
37
37
25
25
25
20
20
20
15
15
15
Contactin-2
GluA1
L1CAM
kDa
kDa
250
250
150
150
100
100
75
75
50
50
37
MAG
25
20
Synaptophysin
Supplementary Figure S2. Extended images of western blotting in this manuscript.
